# Establishment of functional epithelial organoids from human lacrimal glands

**DOI:** 10.1186/s13287-021-02133-y

**Published:** 2021-04-21

**Authors:** Sang Yun Jeong, Woo Hee Choi, Seong Gyeong Jeon, Sookon Lee, Jong-Moon Park, Mira Park, Hookeun Lee, Helen Lew, Jongman Yoo

**Affiliations:** 1 Department of Microbiology and CHA Organoid Research Center, CHA University School of Medicine, 335 Pangyo-ro, Bundang-gu, Seongnam-si, Gyeonggi-do 13488 Republic of Korea; 2 ORGANOIDSCIENCES, Ltd., Seongnam, Gyeonggi-do 13488 Republic of Korea; 3 Department of Rheumatology, Bundang CHA Medical Center, CHA University, Seongnam, Gyeonggi-do Republic of Korea; 4 Department of Pharmacology, Gacheon University, Incheon, Gyeonggi-do Republic of Korea; 5 Department of Ophthalmology, Bundang CHA Medical Center, CHA University, Bundang-gu, Seongnam-si, Gyeonggi-do 13496 Republic of Korea

**Keywords:** Organoid, Lacrimal gland, Dry eye disease, Sjogren syndrome

## Abstract

**Background:**

Tear deficiency due to lacrimal gland (LG) dysfunction is one of the major causes of dry eye disease (DED). Therefore, LG stem cell-based therapies have been extensively reported to regenerate injured lacrimal tissue; however, the number of stem cells in the LG tissue is low, and 2D long-term cultivation reduces the differentiation capacity of stem cells. Nevertheless, 3D LG organoids could be an alternative for a DED therapy because it is capable of prolonged growth while maintaining the characteristics of the LG tissue. Here, we report the development of LG organoids and their application as cell therapeutics.

**Methods:**

Digested cells from human LG tissue were mixed with Matrigel and cultured in five different media modified from human prostate/salivary organoid culture media. After organoid formation, the growth, specific marker expression, and histological characteristics were analyzed to authenticate the formation of LG organoids. The secretory function of LG organoids was confirmed  through calcium influx or proteomics analysis after pilocarpine treatment. To explore the curability of the developed organoids, mouse-derived LG organoids were fabricated and transplanted into the lacrimal tissue of a mouse model of DED.

**Results:**

The histological features and specific marker expression of LG organoids were similar to those of normal LG tissue. In the pilocarpine-treated LG organoid, levels of internal Ca^2+^ ions and β-hexosaminidase, a lysosomal protein in tear fluid, were increased. In addition, the secreted proteins from pilocarpine-treated lacrimal organoids were identified through proteomics. More than 70% of the identified proteins were proven to exosome through gene ontology analysis. These results indicate that our developed organoid was pilocarpine reactive, demonstrating the function of LG. Additionally, we developed LG organoids from patients with Sjogren’s syndrome patients (SS) and confirmed that their histological features were similar to those of SS-derived LG tissue. Finally, we confirmed that the mouse LG organoids were well engrafted in the lacrimal tissue two weeks after transplantation.

**Conclusion:**

This study demonstrates that the established LG organoids resemble the characteristics of normal LG tissue and may be used as a therapy for patients with DED.

**Supplementary Information:**

The online version contains supplementary material available at 10.1186/s13287-021-02133-y.

## Background

Dry eye disease (DED) is a continuously disabling disease occurring in 11%–22% of population, with about 17% of them developing water-deficient dry eyes [1, 2]. A previous study has reported that a severe type of DED occurs in about half of the patients, presenting structural and functional damage in the lacrimal gland (LG) [3]. DED is a multifactorial life-long debilitating disorder mainly caused by functional disturbances in the LG [4]. Causes of glandular dysfunction range from deficiency and loss of tear film integrity, LG deterioration, to death of the secretory epithelial cells affected by hormonal imbalance, environmental changes, and autoimmune pathologies, leading to DED, a chronic condition [4].

Furthermore, DED has also been defined as a multifactorial disease of the ocular surface characterized by the lack of tear film stability. It is accompanied by ocular symptoms in which unstable tear film, hyperosmolarity, ocular surface inflammation and damage, and neurosensory abnormalities play pathological roles [5]. Its etiologies include causes secondary to systemic autoimmune disorders such as Sjogren’s syndrome (SS), rheumatoid arthritis, and systemic lupus erythematous, which are bothersome to doctors and patients [6–10]. In conventional treatment, artificial tear eye drops are mainly used to moisturize the ocular surface and offer additional lubrication [11]. To address chronic inflammation symptoms, anti-inflammatory and topical immunosuppressive agents could be considered [12]. However, their persistent use is limited due to side effects [5, 12]. Other regenerative strategies have recently been introduced to improve DED management [13].

Interestingly, recent studies have demonstrated the habitation of stem cells in exocrine glands such as salivary [14], pancreatic [15, 16], prostate [17], and mammary glands [18, 19]. However, reports on the existence of stem cells in the mouse LG [20, 21] and human LGs are fewer than those in other exocrine glands.

In contrast to LG cultures growing as a monolayer in the order of preference on Matrigel, collagen, and HAM in two to three weeks, the formation of spheroids including a mixed population of stem cells and differentiated cells has been reported in salivary gland cultures [22] and prostate spheres [23]. A single study has been conducted on spheroidal aggregation of rabbit LG cells grown in the microgravity environment of a rotary cell culture system [24]. Salivary spheres including stem cells, can rescue gland function, when they are transplanted into radiation-induced dry mouth animal models [22].

To the best of our knowledge, there are few reports of organoid establishment using stem cells in the human LG. Under certain culture conditions, suspension 3D cultures of human “lacrispheres” are maintained and propagated for three to four weeks. In addition, lacrimal spheres can secrete quantifiable levels of tear protein into conditioned media [23]. LG epithelial cells can form “spherules” with channel-like connections. The secretory lgA, lysozyme, and lactoferrin levels have explained the ductal origin in conditioned media [4].

The structural and functional loss of the LG may be managed by gland replacement and function restoration through cell therapy [23]. Organoid-based therapy containing stem cells would help recovery and differentiation into functionally competent cells, which could lead to damaged tissue regeneration. 

There is increasing evidence for the prevalence of stem-like cells in the mouse LG [21, 25] that contribute to the reconstruction of the injured gland. We attempted to establish cultures of human LG organoids and evaluated stem cell components by immunophenotyping, clonal assays, and real-time functional assays. We aimed to rebuild an organoid presenting a 3D LG tissue functional unit incorporating different cell types. It could provide a premise of treatment options for severe DED models, including SS.

## Methods

### Cell isolation and organoid formation from human lacrimal gland tissue

Normal LG tissues were obtained from non-damaged regions of the patients with eye-related disease. The tissues were chopped and washed with advanced DMEM/F12 (Gibco, Carlsbad, CA, USA) supplemented with 1% penicillin-streptomycin (Welgene, Gyeongsan-si, Korea) and then enzymatically digested with advanced DMEM/F12 supplemented with 0.125 mg/mL dispase II (Wako, Richmond, VA, USA), 0.1 mg/mL DNase I (MilliporeSigma, Burlington, MA, USA), 0.125 mg/ml collagenase II (Gibco, Carlsbad, CA, USA), and 1% penicillin-streptomycin for 1 h at 37 °C with shaking (150 rpm). After digestion, the supernatant was passed through a 70-μm cell strainer (SPL, Pocheon-si, Gyeonggi-do, Korea) and pelleted by centrifugation at 200g for 5 min. The pellet was resuspended in culture media and mixed with Matrigel (Corning, Corning, NY, USA) at a ratio of 1:1 (v:v), plated on a 48-well plate at a density of 1X10^4^ per well, and incubated with 5% CO_2_ at 37 °C for 10 min to polymerize the matrices. The LG organoids were cultured in five different media modified from human prostate and salivary organoid culture medium [26, 27]. The components of each medium are listed in Supplementary Table 1. The culture medium was changed every two to three days.

To confirm the origin of cells forming the organoid, epithelial cell adhesion molecule (EpCAM)-positive epithelial lineage cells were sorted using the MACS method (Miltenyibiotec, USA, 130-042-201). Briefly, single cells from the lacrimal tissue were incubated with anti-EpCAM (Santacruz, CA, USA, sc-59906) for 1 h at 4 °C, washed with MACS buffer, and then incubated with anti-mouse IgG Microbeads (Miltenyibiotec, 130-048-402) for 30 min at 4 °C. After washing with PBS, the EpCAM-negative cells were passed through an MACS column, while the EpCAM-positive cells in the MACS column were isolated, washed, and then cultured in Matrigel.

### Histology and immunofluorescence

Tissues and organoids were washed with D-PBS (Welgene, Korea), fixed with 4% paraformaldehyde (Bio-solution, Seoul, Korea) for 30 min, and embedded in paraffin. Paraffin sections (6-μm-thick) were deparaffinized in xylene and hydrated in a graded ethanol series. The samples were then stained with H&E, Alcian blue, PAS staining kit (Abcam, Cambridge, MA, USA), and Masson’s trichrome staining kit (Dako, Santa Clara, CA, USA) according to the manufacturers’ protocol. For immunofluorescence analysis, fixed samples were cryoprotected by immersion in PBS supplemented with 30% sucrose and 0.1% sodium azide at 4 °C. The cryoprotected samples were embedded in optimal cutting temperature (OCT, Sakura, Japan) compound, rapidly frozen in liquid nitrogen, and stored at − 80 °C until use. Sections (4-μm-thick) of the frozen block were pre-blocked with 5% normal horse serum (Vector, IL, USA) in Tris-buffered saline (Welgene) for 2 h at room temperature (RT) and incubated with primary antibody at 4 °C overnight. After washing with PBS, the sections were incubated with secondary antibody for 2 h at RT. For nuclear staining, the sections were treated with 1 ug/ml Hoechst 33342 (MilliporeSigma, Burlington, MA, USA, 1 μg/ml) for 20 min. Primary antibodies used for immunostaining included antibodies against aquaporin 5 (AQP5; Abcam), alpha-smooth muscle actin (α-SMA; Biolegend, San Diego, CA, USA), vimentin (VIM; Cell signaling, Danvers, MA, USA), lysozyme (LYZ; Diagnostic biosystems, Pleasanton, CA, USA), E-cadherin (E-CAD; Santa Cruz Biotechnology, Dallas, TX, USA), anti-BrdU (Novus, Centennial, CO, USA), and Ki67 (Abcam, Cambridge, MA, USA). The secondary antibodies (Thermo Fisher Scientific, Waltham, MA, USA) used included Alexa Fluor 488 goat anti-rabbit IgG, Alexa Fluor 594 goat anti-mouse IgG, and Alexa Fluor 594 goat anti-rat IgG.

### Total RNA isolation and quantitative RT-PCR

Total RNA was extracted from isolated tissues or organoids using MagListo™ 5 M Cell Total RNA Extraction Kit (Bioneer, Daejeon Metropolitan City, Korea) following the manufacturer’s protocol. Thereafter, 1 μg of RNA was used to synthesize cDNA using PrimeScript™ RT Master Mix (TaKaRa, Kyoto City, Japan). Quantitative RT-PCR was performed with a Thermal Cycler Dice® Real-Time System III (TaKaRa, Kyoto City, Japan) using SYBR® Premix Ex Taq™ II (TaKaRa). The PCR primers sequences are listed in Supplementary Table 2. PCR experiments were carried out in triplicates.

### Calcium flux assay with Fluo-4

Ca^2+^ mobilization to the cytoplasm was detected using a Fluo-4 Calcium Imaging Kit (Thermo Fisher Scientific) following the manufacturer’s protocol. Briefly, the organoids were treated with Fluo-4 AM for 15 min at 37 °C, followed by incubation at RT for 15 min. After washing with PBS, the organoids were stimulated with 1 μg/mL pilocarpine (MilliporeSigma). Calcium signaling was then observed using a Nikon Eclipse Ti2 microscope (Nikon, Tokyo, Japan).

### β-Hexosaminidase assay

To demonstrate the secretory function of LG organoids, the lysosomal enzyme N-acetyl-β-glucosaminidase (NAG), also known as α-galactosidase B, in organoid cultured medium was detected using an NAG assay kit (MilliporeSigma) following the manufacturer’s protocol. Briefly, organoids were incubated in serum-free DMEM/F12 for 2 h, treated with pilocarpine (1 μg/mL), and then incubated at 37 °C in a 5% CO_2_ incubator for 24 h. The medium was collected at 2 h and 24 h after pilocarpine treatment and analyzed for NAG catalytic activity. The reaction product was detected colorimetrically at 405 nm using a microplate reader (Multiskan GO, Thermo Fisher Scientific).

### Transmission electron microscopy (TEM)

The secretory proteins from organoids were detected using TEM . Briefly, the cultured organoids were washed with D-PBS and fixed with 2% glutaraldehyde-paraformaldehyde in 0.1 M phosphate buffer (PB, pH 7.4) for 12 h. After washing with 0.1 M PB, samples were post-fixed with 1% OsO4 dissolved in 0.1 M PB for 2 h, dehydrated in an ascending gradual ethanol series (50%–100%), infiltrated with propylene oxide, and embedded with a Poly/Bed 812 kit (Polysciences, PA, USA). After pure fresh resin embedding and polymerization at 65 °C in an electron microscope oven (DOSAKA, Japan) for 24 h, the Poly/Bed embedded samples were cut into approximately 70-nm-thick sections with a Leica EM UC-7 (Leica Microsystems, Wetzlar, Germany) equipped with a diamond knife (Diatome, PA, USA), transferred to copper and nickel grids, contrast-stained with 6% uranyl acetate and lead citrate (Fisher), and observed using a transmission electron microscope (JEOL, Japan) at 80 kV acceleration voltage.

### Proteomic analysis of LG organoids secretome

To identify the proteins secreted by LG organoids after pilocarpine treatment, the culture medium was harvested 2 h after of pilocarpine treatment and analyzed through proteomics analysis [28]. In brief, proteins (200 μg) in the medium were digested using the filter-aided sample preparation (FASP) method with centrifugal filters (Millipore, MA, USA). After desalting the samples with a Sep-Pak® Vac 1 cc C18 cartridge (Waters, MA, USA), the peptides were collected, purified, and quantified via LC-MS/MS analysis. LC-MS/MS assay was performed using a Dionex Ultimate 3000 HPLC coupled with a Q Exactive™ Hybrid Quadrupole-Orbitrap mass spectrometer (Thermo Fisher Scientific, Waltham, MA, USA). Raw MS/MS data were quantified using MaxQuant (Max Planck Institute) and classified by gene ontology (GO) analysis. T test *P* < 0.05 and fold-change (> 2, < − 2) were applied to determine the differentially expressed proteins (DEPs) between the control and pilocarpine-treated groups.

### Mouse dry eye model and organoid transplantation

Eight-week-old male C57BL/6 mice (Koatech, Pyeongtaek, Korea) or C57BL/6-Tg (CAG-EGFP)131Osb/LeySopJ mice (Nihon SLC, Shizuoka, Japan) mice were used as DED models or for the manipulation of LG organoids, respectively. The experimental protocol for animal use was reviewed and approved by the CHA University Institutional Animal Care and Use Committee. The LG tissue was obtained from eGFP-Tg mice. Organoids were formed and cultured following the same method used for human organoids. To create an inflammation-induced dry eye model, 15 μL concanavalin A (ConA, 10 mg/mL in PBS, MilliporeSigma) was injected into the extra-orbital gland of wild-type mouse lacrimal tissue. The same volume of PBS was injected into the control group. Seven days after ConA injection, cell clumps from GFP-expressing organoids (1 × 10^4^ cells/15 μL in advanced DMEM/F12/Matrigel) were injected into the extra-orbital gland space (Fig. 5). After two weeks, the mouse LG tissues were harvested for immunofluorescence analysis.

### Statistical analysis

Statistically significant differences were analyzed by Student’s *t* test of one-way analysis of variance (ANOVA) with post hoc Tukey test for multiple comparisons using the GraphPad Prism software package, version 3.0 (GraphPad Prism, CA, USA). All experiments were conducted at least thrice. The number of independent experiments is indicated by n. Significance was considered at *p* < 0.05.

## Results

### Generation of LG organoids from human tissues

**Fig. 1:**
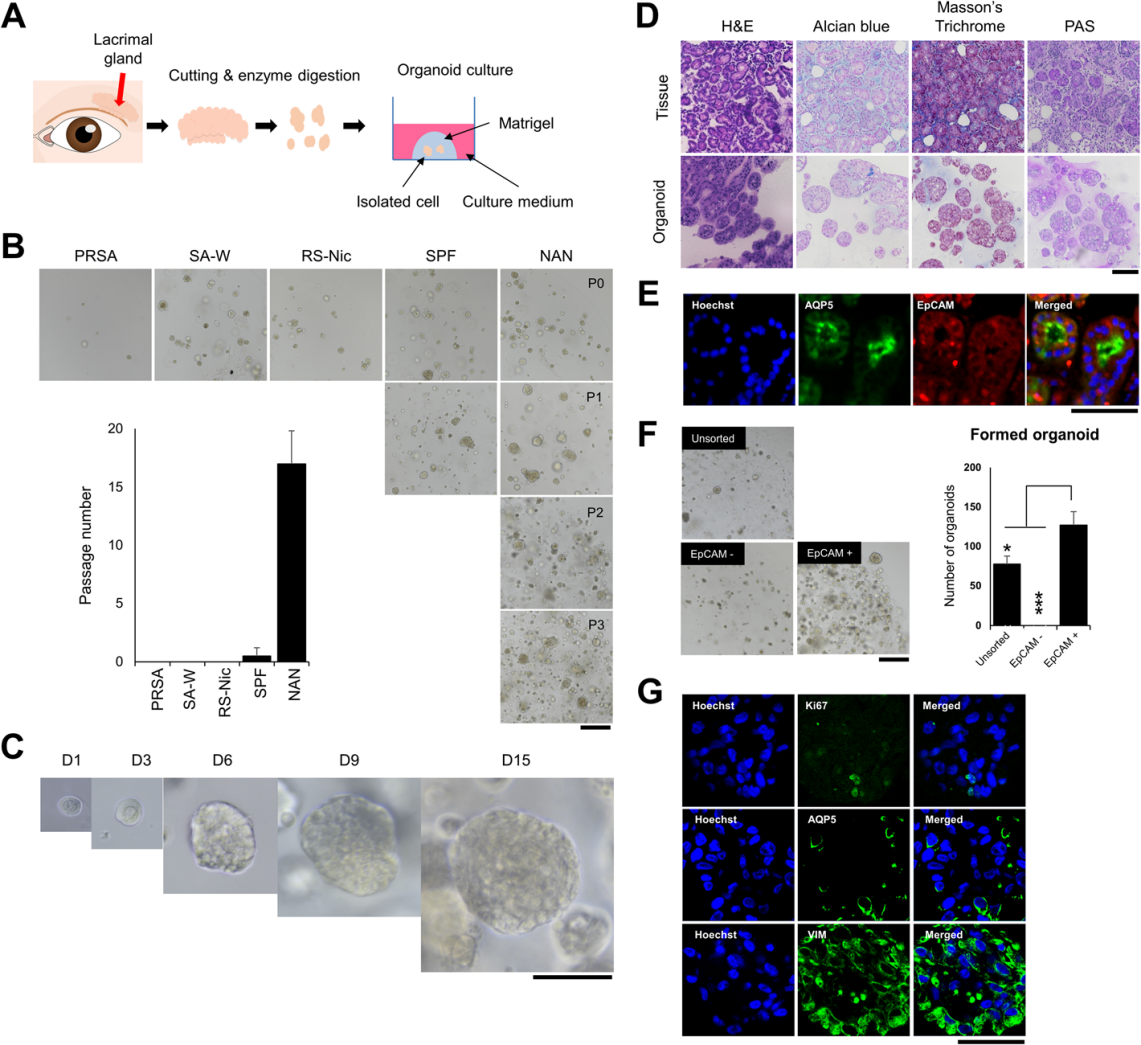
Generation of epithelial organoids from human lacrimal glands (LGs). **A** Scheme for the development and culture of lacrimal tissue-derived organoid. **B** Optimization of culture media following organoid formation and subculture. Representative images were obtained by light microscopy after culturing for 3 days (Magnification; 10×, Scale bar; 500 µm). **C** The growth of organoid cultured in optimized LG organoid medium (LGOM). Scale 100 μm. **D** A comparison of histological analysis between normal lacrimal tissue and formed LG organoid. H&E, Alcian blue, Masson’s trichrome, and PAS staining were performed for identifying the acini structure, mucosubstance secretion, connective tissue, and glycoprotein secretion, respectively (Magnification; 40×, Scale bar; 100 µm). **E** Confirmation of acinar cells (green; aquaporin 5; AQP5) and epithelial lineage (red; EpCAM) cells in lacrimal tissue (Magnification; 100×, Scale bar; 50 µm). **F** Organoid formation from EpCAM-positive cell fraction. Representative bright-field images (Magnification; 10×, Scale bar; 500 µm) were obtained after 4 days of cultivation and used for counting the formed organoid (right, graph, *N* = 4). **G** Ki67, AQP5, and vimentin (VIM) expression was detected in the organoids and observed using immunofluorescence (Magnification; 100×, Scale bar; 50 µm)

To generate LG organoids, we used human LG tissues and allowed them to self-organize within Matrigel (Fig. 1A). The dissociated LG cells were embedded in Matrigel and then grown respectively in five different conditions by modifying human prostate or salivary gland organoid media. Most organoids were generated under all medium conditions. In the M-SA1 medium (salivary gland organoid media supplemented with 10 mM nicotinamide, 500 nM A83, and 100 ng/mL noggin); however, the formation and growth of organoids were maintained following passage, unlike that in other media. Therefore, we defined M-SA1 medium as an LG organoid medium (LGOM); (Fig. 1B). In the LGOM, LG organoids expanded until passage 19 (Fig. 1B) and increased in size until day 15 (Fig. 1C). Histological analysis showed that the organoids were similar to the LG  tissues. Acinar cells were the major cell type in the LG tissue, and its secreted acidic mucosubstances were observed (Fig. 1D, H&E and Alcian blue staining, respectively). Secretory products such as glycogen and glycoprotein were also observed in normal lacrimal tissue and organoids (Fig. 1D, PAS staining). Masson’s trichrome staining showed that keratin was the major ECM component of organoids (Fig. 1D). These results showed that the organoids developed in this study were morphologically similar to the acinar cells of the LG tissue.

Additionally, we confirmed the expression of EpCAM, an epithelial lineage cell marker, in the lacrimal tissue (Fig. 1E). EpCAM-positive cells generated more LG organoids than a non-sorted fraction, whereas organoids were not formed in the EpCAM-negative cell fraction (Fig. 1F). A large amount of VIM, a gland-specific marker, was observed in the organoids developed from EpCAM-positive cells. These results showed that the organoids originated from EpCAM-positive cells (Fig. 1G).

### Human LG organoids recapitulate the structural properties of human tissues

**Fig. 2:**
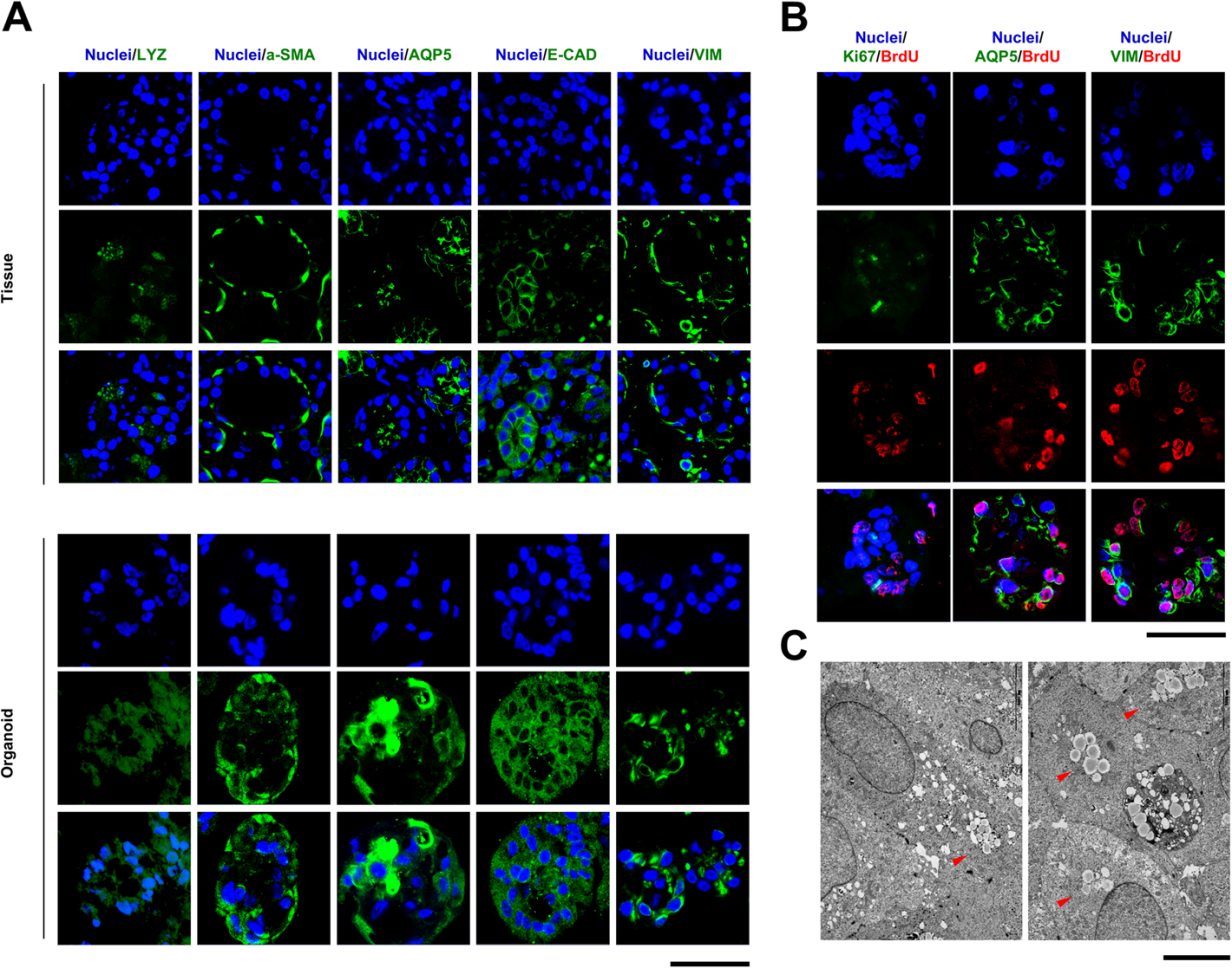
Structural recapitulation of human lacrimal gland (LG) organoids. **A** Immunofluorescence staining for specific Markers, such as vimentin (VIM), E-cadherin (E-CAD), aquaporin5 (AQP5), alpha-smooth muscle actin (α-SMA), and lysozyme (LYZ), was performed in human LG tissue and organoid cultured in LG organoid medium (LGOM). **B** In the organoid, the proliferating cells were confirmed via BrdU assay. At 4 days after BrdU treatment, proliferating cell (BrdU and Ki76) and LG-specific (AQP5 and VIM) markers) were detected in organoids (Magnification; 100×, Scale bar; 50 µm). **C** TEM analysis was performed to confirm cellular ultrastructure in the organoid. Acinar cells (up; white dotted line) and secretory granules (down; red arrowhead) were detected in the LG organoid (Scale bar; 5 µm)

The developed LG organoids were compared with human LG tissues. Specific markers such as AQP5, LYZ, E-CAD, VIM, and α-SMA were used for immunofluorescence staining (Fig. 2A). VIM, AQP5, and LYZ expression patterns in organoids were similar to those in acinar cells of LG tissues. Additionally, E-CAD and α-SMA expression patterns in LG organoids were similar to those in the tissues, although their expression levels were lower than those in lacrimal tissues. These markers are known to be expressed in acinar cells, but not in ductal cells. Therefore, the organoids formed in this study are more similar to acinar cells than to ductal cells in lacrimal tissues. Additionally, we confirmed the proliferating cells in the LG organoid using the BrdU assay (Fig. 2B). Four days after BrdU treatment, the BrdU-positive cells expressed Ki67 in the outer region of organoids, unlike the inner region. In particular, VIM expression was observed around BrdU-positive cells.

To determine the cellular ultrastructure, LG organoids were analyzed via TEM. In some organoids, cells (Fig. 2C, white dotted line) with secretory granules (Fig. 2C, black arrowhead) were observed in organoids. These characteristics are known to be the features of acinar cells.

### Human LG organoids recapitulate the structural properties of human LGs

**Fig. 3:**
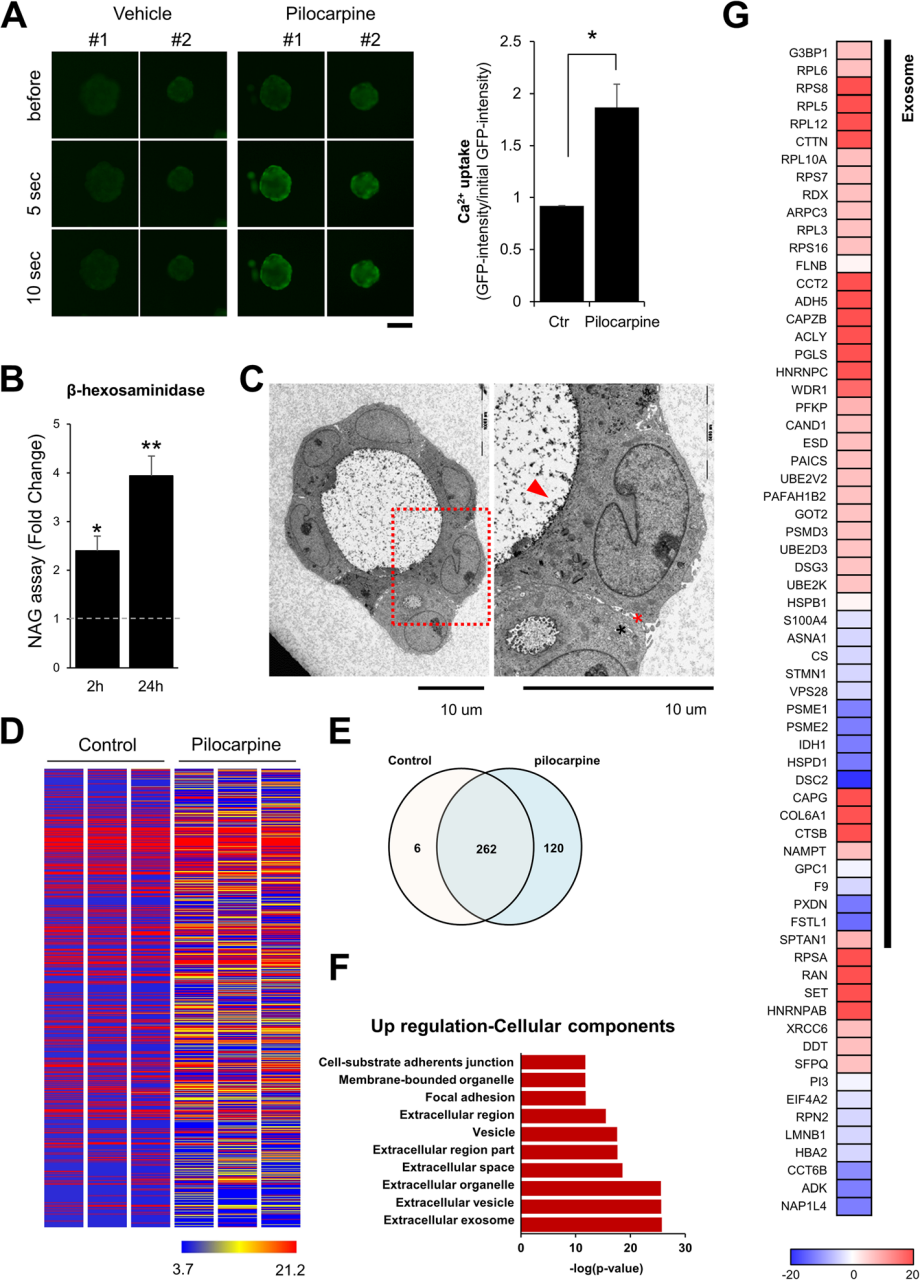
Functional recapitulation of human lacrimal gland (LG) organoids. To confirm the secretory function of the organoid, **A** Ca^2+^ uptake (Magnification; 10×, Scale bar; 100 µm) and **B** released β-hexosaminidase was detected after 1 μg/mL pilocarpine treatment, and **C** the secretome released from the organoid was observed via TEM. Proteins secreted by pilocarpine were identified using proteomic analysis. Through the **D** heatmap, **E** Venn diagram, **F** gene ontology (GO) term analysis, and identification of the differentially expressed proteins (DEPs), the proteins were seen to be more secreted from pilocarpine-treated organoids than from the normal organoids, and most proteins belonged to exosome/vehicle

In this study, we confirmed the secretory function of developed organoids by pilocarpine stimulation. After stimulation with 1 μg/mL pilocarpine, Ca^2+^ concentration was increased in the cells of organoids (Fig. 3A). In the cells of the LG, increasing Ca^2+^ concentration are known to lead to tear secretion [29]. Additionally, secretion of β-hexaminidase, a lysosomal enzyme, was increased in pilocarpine-treated organoids (Fig. 3B). In particular, the secreted proteins were observed (Fig. 3C, red arrowhead), and cell-cell junction was widened (Fig. 3C, asterisk) in pilocarpine-treated organoids. These results indicate that the organoids developed in the present study have a secretory function in response to the cholinergic agonist pilocarpine, similar to the LG tissue.

### Human LG organoids recapitulate the functional properties of human LGs

To identify the proteins secreted by the LG organoids after pilocarpine treatment, the culture medium was collected and analyzed for proteomics after 2 h after PBS or pilocarpine treatment. A total of 776 proteins were identified and quantified in both groups, and the amount of the proteins was additionally secreted after pilocarpine treatment (Fig. 3D-E). In the pilocarpine-treated organoid, the upregulated cellular components were found to be extracellular exosome/vehicles by GO analysis (Fig. 3E-F). Additionally, we identified 66 DEPs (fold-change >2), and most proteins (>70%) belonged to exosome/vehicle (Fig. 3G). Among the upregulated proteins in the pilocarpine-treated group, the protein synthesis-related secretome (such as RPS and RPL family) was found to be increased. These results indicate that our developed lacrimal organoids might turn on synthesis processes such as tear production by the stimulator.

### Generation of LG organoids derived from LGs of the patients with SS

**Fig. 4:**
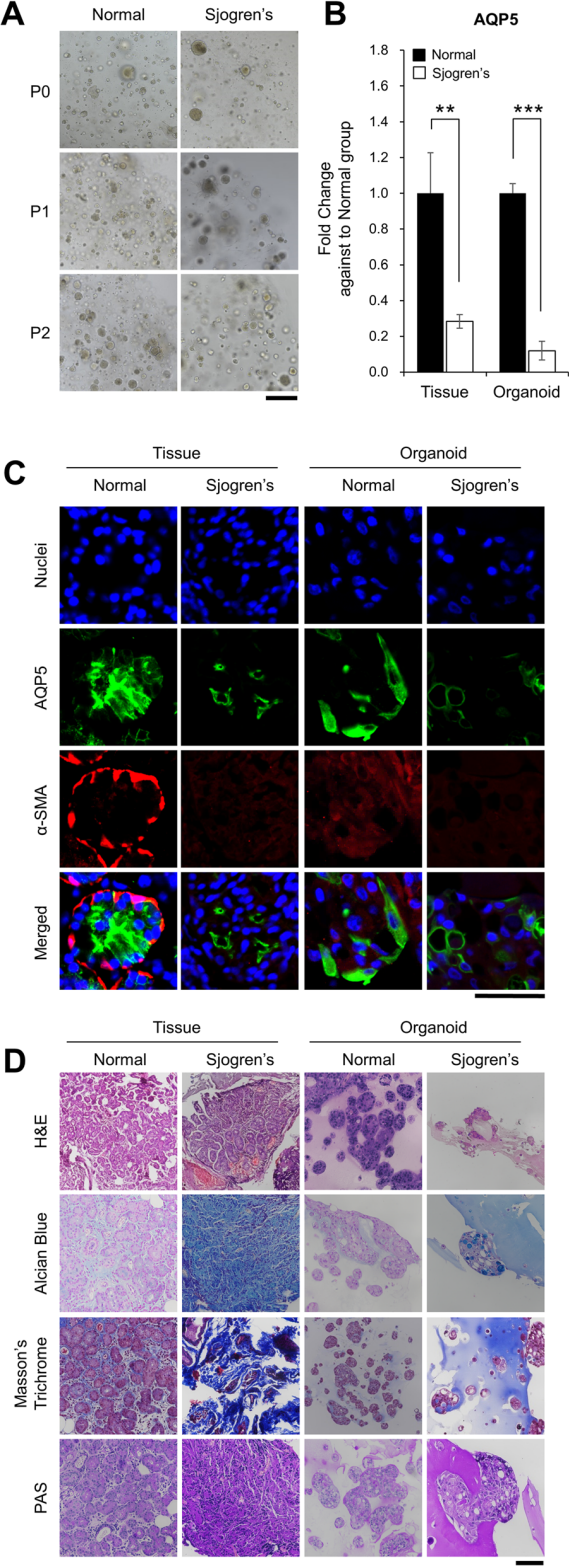
Generation of lacrimal gland (LG) organoid from patients with Sjogren’s syndrome (SS). **A** Images of formed SS organoids were obtained by light microscopy after culturing for 3 days (Magnification; 10×, Scale bar; 500 µm). **B** Gene expression of AQP5, the LG-specific marker, was analyzed by qRT-PCR (*N* = 3). **C**, **D** The SS LG lacrimal gland tissue and organoids were compared with normal lacrimal tissue and organoids by immunofluorescence (Magnification; 100×, Scale bar; 50 µm) and histological analysis (Magnification; 40×, Scale bar; 100 µm)

To advance our research, we developed LG organoids from the tissue of patients with SS, which are referred to as SS organoids. SS organoids were generated and cultured following the same method used for normal organoids. SS organoids featured a smaller size and were cultured for shorter passages (up to passage 2), as compared to normal organoids (Fig. 4A). Expression of AQP5, an LG-specific marker, was significantly lesser in the SS tissue and organoids than that in the normal tissue and organoids (Fig. 4B, C). Moreover, histological analysis revealed that SS organoids have higher fibroblastic properties than the normal tissue and organoids, while their degree is similar to that of SS tissue (Fig. 4D). Morphologically, some structural damages in acinar cells were found through H&E analysis and, dense blue regions representing collagen-stained areas were observed after Masson’s trichrome staining of SS organoids, similar to the SS tissue, whereas the normal organoid showed an intact structure in the light blue area, as in the normal tissue. These results suggest that our developed SS organoids could be a useful tool as an *in vitro* model of mimetic SS disease.

### Engraftment of LG organoids in the DED mouse model 

**Fig. 5:**
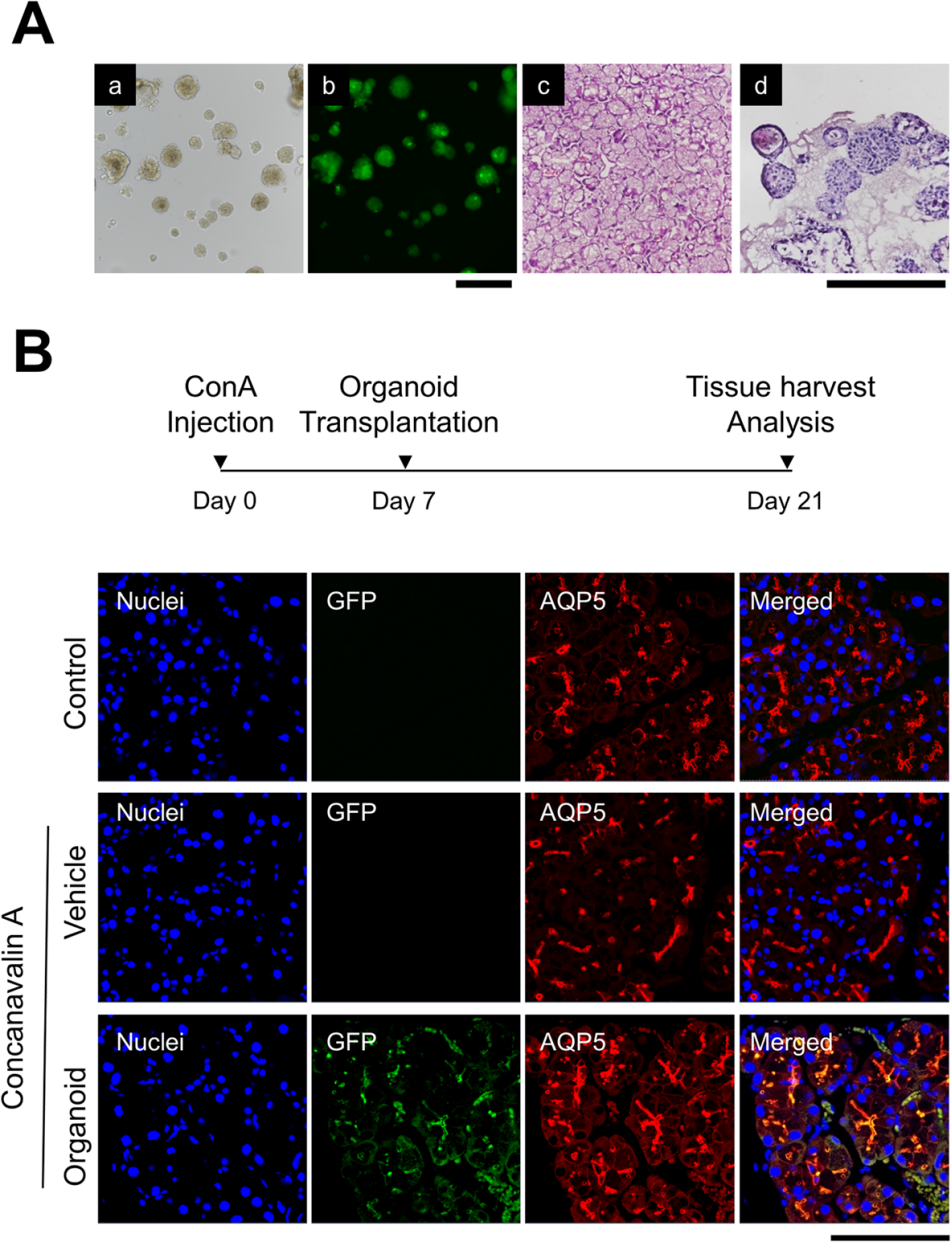
Engraftment of lacrimal gland (LG) organoid into dry eye mouse model. **A** (a–b) Mouse lacrimal organoids were formed from lacrimal tissue of C57BL/6-GFP-Tg mice. The GFP signals were expressed in the formed organoid (Magnification; 10×, Scale bar; 500 µm) and **A** (c–d) their morphology was compared to that of mouse LG tissue through H&E staining (Magnification; 20×, Scale bar; 500 µm). **B** Inflammation-based dry eye disease (DED) animal model was established by concanavalin A (ConA) injection into the extra-orbital gland of the mouse. GFP-labeled organoids were transplanted into mouse lacrimal tissue after 7 days after ConA injection, and their engraftment was confirmed by GFP signal after 14 days after transplantation. Expression of aquaporin 5 (AQP5 ) was analyzed by immunofluorescence (Magnification; 40×, Scale bar; 100 µm)

To confirm the engraftment ability of LG organoids, the organoids from the LG tissue of C57BL/6-GFP-Tg mice were prepared following the method used for human organoids. GFP-labeled organoids were formed (Fig. 5A, a–b), and their morphology was similar to that of the acinar cells of the mouse lacrimal tissue (Fig. 5A, c–d). In this study, we established an inflammation-based DED mouse model by injecting ConA into the extra-orbital gland of the lacrimal tissue and transplanting the formed GFP-organoids into the lacrimal tissue of the DED mouse. GFP signals were observed in mouse lacrimal tissue after 14 days after transplantation (Fig. 5B). Additionally, we confirmed that AQP5, a water channel protein for tear production, was expressed in the transplanted organoids (Fig. 5B). These results suggest the therapeutic potential of LG organoids for the regeneration of DED due to the injured lacrimal tissue.

## Discussion

The LG plays an essential role in lubricating the ocular surface epithelium, similar to other exocrine glands of the body [4]. Both lacrimal and salivary gland acinar cells share developmental, morphological, and functional characteristics. Additionally, they share a similar reduction in function when injured [30, 31]. A preliminary report has shown that stem cells in the mouse LG can be cultured *in vitro* [21]. Studies have shown that  stem/progenitor cell compartments are present in the salivary gland terminal ductule [14]. This proves that human LG cells can be cultured *in vitro* and can retain their secretory function. In addition to the presence of differentiated cells (epithelial, myoepithelial, and stromal cells), we recapitulated the real gland as an “organoid” with typical cell-to-cell junctional connections.

Previously, simulated microgravity has been shown to promote the development of spheroidal aggregates of rabbit LG cells with a mean diameter of 384.6 ± 111.8 μm after 7 days [24]. These spheroids consist of organized LG cells and acini-like structures. The mean diameter of these spheroids is maintained continuously over a culture period of approximately 28 days. However, apoptosis occurs at the center of spheroid aggregates, correlating with spheroid diameter and culture period duration (between days 14 and 21 and between days 21 and 28) [24]. The culture conditions in this study provided a stable environment for maintaining the size and integrity of the organoid grown from human LG stem cells.

Another method to differentiate iPS cells into an LG epithelial cell phenotype would require a development process, although this has already been described for embryonic stem cells [25]. Spheroids can be formed by assembling three cell types: epithelial cells, MSCs isolated from a porcine LGs, and endothelial cells isolated from human foreskin [32]. They retain their own immunophenotypes with spontaneous organization in response to parasympathetic stimulation, although spheroid function, cell proliferation, and viability decrease with apoptosis over time [32]. This study demonstrated the possibility of constructing a true LG functional unit, comprising lacrimal epithelial cells and MSCs from adult mammalian tissues *in vitro*.

Much effort has been put into the culture of the LG *in vitro*. For example, experiments in a micrograft environment and the transfer of amniotic membranes have been performed. However, these methods have not shown expected results over an extended duration [33]. We explored five media by modifying the salivary gland organoid media and applied them to organoid culture. We finally concluded that M-SA1 medium is the best LGOM because it resulted in the longest surface, maximum length of organoids formed, and the best expansion. Similarly, murine LG stem cells can express stemness markers (such as Nanog, Sox2, and Klf4), known as early lineage markers of all three germ layers [33]. In this study, LG organoids from normal individuals presented gland-specific markers such as VIM, E-CAD, AQP5, and α-SMA, although their expression was lower than that in the tissue. As shown in Fig. 4, the expression pattern of specific markers in organoids from normal and patients with SS reflects the difference between the two tissues. Patients with SS, who produce less tear secretion than a normal person, cannot participate in the Ca^2+^ signaling pathway, as they do not have any Ca^2+^ channel in the myoepithelial cell plasma membrane [34]. Myoepithelial cells were altered in the patients with SS and α-SMA from that in normal individuals. These results suggest that our developed SS organoids could be a useful tool for *in vitro* model of mimetic SS.

In this study, we tried to confirm the secretory function of the LG organoid and measured its secretory function *in vitro*. Muscarinic receptors should be activated by various stimulants to induce tear secretion from the LG. Under these conditions, internal Ca^2+^ increase leads to tear secretion [35]. Pilocarpine is a parasympathetic stimulant mainly affecting muscarinic M3 receptors. Since organoids do not have nerves, they are to be stimulated using pilocarpine, an M3 receptor agonist. Therefore, internal Ca^2+^ and β-hexosaminidase, a known lysosomal protein in tear fluid, increased in the pilocarpine-treated organoid. In addition, the secreted proteins from pilocarpine-treated lacrimal organoids were identified through proteomics. These results indicate that our developed organoid is pilocarpine reactive, demonstrating the function of the LG.

Moreover, this study preliminary demonstrated that the LG organoid possessed a self-repair effect of stem cells in an inflammation-induced DED animal model. However, its future role merits further investigation. Although DED has a moderate prevalence globally, primary clinical management is still conservative using artificial tear drops and lubricants [4]. Transplanted MSCs are well known to enhance corneal wound healing by trophic factor production and immune regulatory effects instead of direct transdifferentiation into epithelial cells [36–39]. As cell therapy prerequisites, the plausibility of using lacrimal organoids containing functional stem cells to rescue and repair functionally competent cells should be studied. Such studies may contribute to damaged tissue regeneration [23].

We tried to determine the possibility of regenerating the damaged LGs using lacrimal organoids after inducing inflammation using ConA in C57BL/6 mice. We hypothesize that damages to the LG due to aging, hormonal imbalance, or radiation may be treated using organoids. In summary, this study provided the first evidence for successful growth of fresh human LG organoids *in vitro* with an attempt toward functional unit formation while retaining secretory function. Further validation is needed for the development of a functionally competent secretory lacrimal organoid for potential clinical application in severe DED, including autoimmune LG disorders.

## Conclusion

In conclusion, we established lacrimal organoids from human and mouse lacrimal tissues. The organoids established in this study recapitulate the structure and function of human LGs, suggesting that organoids can be used as a tool for disease modeling and regenerative therapy.

## Supplementary Information

**Additional file 1: Table S1.** The components of culture medium for lacrimal gland organoid. **Table S2.** The primers used for quantitative RT-PCR.

## Data Availability

The datasets generated during and/or analyzed during the study are available from the corresponding author on reasonable request.

## Abbreviations

DED: Dry eye disease
LGOM: Lacrimal gland organoid medium
PR: Medium for prostate organoid cultivation
SA: Medium for salivary organoid cultivation
M-SA1/M-SA2: Modified from salivary organoid culture medium
M-PRSA: Modified from prostate and salivary organoid culture medium
VIM: Vimentin
E-CAD: E-cadherin
AQP5: Aquaporin5
α-SMA: Alpha-smooth muscle actin
LYZ: Lysozyme
SS: Sjogren’s syndrome
ConA: Concanavalin A
