# Supplementary material for: Establishment of functional epithelial organoids from human lacrimal glands
Source: Stem Cell Res Ther. 2021 Apr 21;12:247. doi: 10.1186/s13287-021-02133-y (PMC8059179; doi:10.1186/s13287-021-02133-y)
Supplement: Supplementary file 1 — Additional file 1: Table S1. The components of culture medium for lacrimal gland organoid. Table S2. The primers used for quantitative RT-PCR. [file 13287_2021_2133_MOESM1_ESM.docx]

**Supplementary Table 1.** The components of culture medium for lacrimal gland organoid.

| **Groups** | **PR** | **SA** | **M-SA1** | **M-SA2** | **M-PRSA** |
| --- | --- | --- | --- | --- | --- |
| conditioned-Wnt3a medium | / | 50% | 50% | 50% | 50% |
| conditioned-R-sponding medium | 10% | 10% | 10% | 10% | 10% |
| FGF2 | 5 ng/ml | 20 ng/ml | 20 ng/ml | 20 ng/ml | 5 ng/ml |
| FGF10 | 10 ng/ml | / | 10 ng/ml | / | 10 ng/ml |
| N2 | / | 1X | 1X | 1X | 1X |
| B27 | 1X | / | / | / | 1X |
| Nicotinamide | 10 mM | / | / | 10 mM | 10 mM |
| A83-01 | 5 µM | / | / | 500nM | 5 µM |
| Noggin | 100 ng/ml | / | / | 100 ng/ml | 100 ng/ml |
| EGF | 25 ng/ml | 20 ng/ml | 20 ng/ml | 20 ng/ml | 25 ng/ml |
| SB202190 | 3 µM | / | 3 µM | / | 3 µM |
| Prostaglandin E2 | 1 µM | / | 1 µM | / | 1 µM |
| Insulin | / | 10 ug/ml | 10 ug/ml | 10 ug/ml | 10 ug/ml |
| Dexametasone | / | 1 µM | 1 µM | 1 µM | 1 µM |
| Y-27632 | / | 10 µM | 10 µM | 10 µM | 10 µM |
| N-Acetyl-L-cysteine | / | 500 µM | 500 µM | 500 µM | 500 µM |

**Abbreviation**; PR (medium for prostate organoid cultivation), SA (medium for salivary organoid cultivation), M-SA1/M-SA2 (modified from salivary organoid culture medium). M-PRSA (modified from prostate and salivary organoid culture medium)

**Supplementary Table 2.** The primers used for quantitative RT-PCR.

| **Gene** | **Strand** | **Primer**  **sequences** | **Annealing Temp.** | **Product**  **size** |
| --- | --- | --- | --- | --- |
| Aquaporin-5 (AQP5) | Forward | CCCGCTCACTGGGTTTTCTG | 60 °C | 181bp |
|  | Reverse | CCATGGTCTTCTTCCGCTCTT |  |  |
| GAPDH | Forward | CAGAAGACTGTGGATGGCCC | 60 °C | 292bp |
|  | Reverse | CCACCTGGTGCTCAGTGTAG |  |  |
